# Costs of Hospital Admission on Primary Immunodeficiency Diseases

**Published:** 2017-03

**Authors:** Kheirollah GHOLAMI, Elaheh LAALI, Hassan ABOLHASSANI, Alireza AHMADVAND, Niayesh MOHEBBI, Mohammad Reza JAVADI, Asghar AGHAMOHAMMADI, Nima REZAEI

**Affiliations:** 1. Dept. of Clinical Pharmacy, School of Pharmacy, Tehran University of Medical Sciences, Tehran, Iran; 2. Research Center for Rational Use of Drugs, Tehran University of Medical Sciences, Tehran, Iran; 3. Research Center for Immunodeficiencies, Children’s Medical Center, Tehran University of Medical Sciences, Tehran, Iran; 4. Primary Immunodeficiency Diseases Network (PIDNet), Universal Scientific Education and Research Network (USERN), Stockholm, Sweden; 5. School of Clinical Sciences, Queensland University of Technology, Brisbane, Australia; 6. Primary Immunodeficiency Diseases Network (PIDNet), Universal Scientific Education and Research Network (USERN), Tehran, Iran; 7. Dept. of Immunology, School of Medicine, Tehran University of Medical Sciences, Tehran, Iran; 8. Network of Immunity in Infection, Malignancy and Autoimmunity (NIIMA), Universal Scientific Education and Research Network (USERN), Tehran, Iran

**Keywords:** Cost of diseases, Primary immunodeficiency, Hospitalization, Medical health care, Drugs

## Abstract

**Background:**

Primary immunodeficiency diseases (PID) are heterogeneous group of inherited disorders mainly characterized by recurrent infections leading to several times hospital admissions. The economic impact of PID is a challenging issue; therefore, this study was designed to determine the medical costs of hospitalizations in this group of patients as an indicator of the direct cost of these diseases.

**Methods:**

One hundred and ten children with PID hospitalized in the Children’s Medical Center Hospital, Tehran, Iran were included in this study during Jan 2011 and Jan 2012. All direct costs during the admission period were calculated, using the hospital information system.

**Results:**

Medical cost was 7.090$ per patient per admission. Among them, about 1.580$ belong to drug consuming during hospitalization. Anti-infective drugs for systemic use were the most cost-consuming group of drugs, followed by alimentary tract and metabolism and blood and blood forming organs agents. Investigation of anti-infective group internally showed that immune sera and immunoglobulin and antiviral agents for systemic use consisting the most important medication for PID patients during hospital admission.

**Conclusion:**

Although the results of economic evaluations in a region cannot necessarily be applied to other regions, having an overall estimation of hospital admission costs and types of drugs used during admission could be helpful in health policy system.

## Introduction

Primary immunodeficiency diseases (PID) are characterized by defects in the immune system, mainly due to single gene defect (1). This heterogeneous, inherited group of diseases led to absent or reduced function in one or more components of the immune system, leading to variable immune-related complications (2).

Recurrent mucosal infections, persistent fungal infection, deep organ abscesses, enteropathy, granulomatous lesions, opportunistic infections, autoimmunity, lymphoproliferative disorders, and malignancies are the most important manifestations of PID patients (3, 4). Early detection, preventing complications, and adequate treatments are the keys to reducing morbidity and mortality of patients (5–7). Although the true prevalence of PID is unknown, it is not considered as a rare condition (8–11). Physician awareness could lead to better identification of diseases. Although prevalence of each type of PID differs from others, ethnicity and consanguinity could change the incidence pattern of disease, especially in those with autosomal recessive inheritance (12, 13).

Patients with PID usually experience several hospital admissions, especially in emergency, immunology, and infectious diseases wards (14, 15). Antibiotic and antifungal agents are the mainstay treatment to control, prevent and manage chronic and recurrent infections. Immunoglobulin replacement therapy both intravenously and subcutaneously is another effective therapy in antibody deficiencies, also recommended for treatment of many patients with other forms of PID associating hypogammaglobulinemia (5). Other therapeutic approaches include cytokine therapy, enzyme replacement, vaccinations and hematopoietic stem cell transplantation (HSCT) (5).

Nowadays, economic analyses importance has been at the center of attention also in health system, predominantly with respect to health policy making for specific diseases and pharmaceutical recourses (16). In this cost-conscious environment, it is important to evaluate exact costs of diseases to choose less therapeutic modalities with high effectiveness to achieve better outcomes (17). This valuable knowledge will illuminate treatment guideline and health budgeting.

One of the most important sections of estimating financial burden of disease is admission cost, which is crucial for proper decision-making process. Evaluation of admission expenditures can assist health care providers into two goals of budget assignment for medication and cares as well as investigating efficiency of different health interventions by means of either cost-benefit or cost-effectiveness analysis (18). Although burden of some special PID disease was measured (19–21), there is no specific data regarding the cost and economic burden of whole PID group.

This study was designed to estimate the admission cost of PID in the main referral hospital.

## Materials and Methods

### Study environment

During the period of study from Jan 2011 to Jan 2012, the patients with diagnosis of PID hospitalized in the Children’s Medical Center Hospital, Tehran, Iran were enrolled in the study. This hospital is the Pediatrics Center of Excellence in Iran affiliated to Tehran University of Medical Sciences, also known as the main referral center for PID patients. The research center for immunodeficiency located in this hospital is the documenting center of European society for Immunodeficiency and PID patients are diagnosed and treated in this center based on the updated standardized guidelines (22). Therefore, the registered costs for the patients admitted to this tertiary center can be a representative for direct economic costs due to hospitalization in-patient with definite PID diagnosis.

The process of this study was approved by the Ethics Committee of the Tehran University of Medical Sciences, Iran and the extracted data were anonymized for further analysis.

### Patient recruitment

The patients were included in the survey, based on the International Classification of Diseases-10 (ICD-10) code, which extracted from Chapter III (Diseases of the blood and blood-forming organs and certain disorders involving the immune mechanism; D50–D89) (23). The following patients categories were included: D83.9: Common variable immunodeficiency; D81.9: Combined immunodeficiency; D80.4: Selective deficiency of IgM; D80.2: Selective deficiency of IgA; D71: Chronic granulomatous disease; D82.4: Hyper IgE syndrome; D70: Agranulocytosis (for just patients with severe congenital neutropenia); D80.0: X-linked agammaglobulinemia; D82.0: Wiskott-Aldrich syndrome; D84.9: Immunodeficiency, unspecified (for other defined PID, not specified in the ICD-10) (24, 25).

### Medical records

The electronic medical records of all patients with PID were extracted from hospital information system (HIS) during the period of study. Collective information on demographic data (gender, age, number of admissions, length of admission, season of admission and ICD code), and variable medications and other health care’s costs of admission were recorded. HIS database record the kind and frequency of hoteling facilities, hospital bed occupancy, medication (e.g. drugs, fluids administered), consumables (e.g. syringes, tubes, and catheters), para-clinical analyses (e.g. laboratory tests, imaging, pathological investigation), and interventional procedures (e.g. surgery, bronchoscopy) were all evaluated in total cost of admission for each individual patient. We categorized all drugs cost into 12 groups, according to anatomical-therapeutic-chemical (ATC) code of World Health Organization (26); then we calculated the cost of each group individually and in different strata of PID. After data extraction, total admission related costs were calculated by addition of all counted costs and converted to US dollar, based on currency exchange in the same period in 2011 as previously described (27).

### Statistical analysis

Statistical analysis was performed using SPSS statistical software package (SPSS Statistics 17.0.0, (Chicago, IL, USA). One-sample Kolmogorov-Smirnov test estimated whether data were normally distributed. Parametric and nonparametric analyses were performed based on the finding of this evaluation. A *P*-value of 0.05 or less was considered statistically significant.

## Results

### Patients’ characteristics

One hundred and ten patients with PID were admitted the Children’s Medical Center in 2011, including 65 males and 45 females. Patients with combined immunodeficiency (27 cases with ICD10: D81.9) were the most frequent type of PID admitted in the hospital (Table 1). The average age of patient at the time of admission was 5.9±3.7 yr, ranging from 19 d to 18 yr old, while average length of admission was 16.33±14.7 d (range of 1 d to 2 months).

**Table 1:** Data analysis on cost of admission in different types of primary immunodeficiency diseases

**ICD 10**	**Disease**	**Number of patients**	**M/F**	**Cost mean**	**SD**	**Drug cost percentage (%)**	**SD**
D83.9	Common variable immunodeficiency, unspecified	18	11/7	2367.0	368.7	11.19	2.2
D81.9	Combined immunodeficiency, unspecified	27	13/14	2096.2.6	499.2	28.1	7.2
D80.4	Selective deficiency of IgM	5	5/0	7675.2	1440.6	9.3	7.1
D80.2	Selective deficiency of IgA	7	5/2	1148.8	1011.8	7.7	4.9
D71	Chronic granulomatous disease	15	12/3	5716.0	1462.2	9.9	6.5
D82.4	Hyper Ig E syndrome	12	2/10	2591.0	663.8	22.4	2.8
D70	Agranulocytosis	8	3/5	2582.2	415.1	11.1	7.8
D80.0	X linked agammaglobulinemia	1	1/0	285	−	4.6	−
D82.0	Wiskott Aldrich syndrome	2	2/0	2327	−	8.7	−
D84.9	Immunodeficiency, unspecified	15	11/4	3925.3	940.2	16.5	9.5

### Admission costs

The mean cost of admission was 7090.4±2663.9$, ranging from 447$ to 256.000$.

The mean cost of admission per day was estimated about 284.7$. Comparison of the cost of admission in different types of PID failed to show any significant difference between the amounts of payments (F=1.30, *P*=0.2).

### Drug costs

The mean cost of drugs and medications was 1579.8±498.6$; 17.2±3.9 percent of total medical costs. The mean cost of drug per day of admission was estimated about 90$.

The greatest mean costs of drug groups belonged to anti-infective for systemic use (770$) which followed by alimentary tract and metabolism (464$) and blood and blood forming organs agents (168$). Investigation of anti-infective group internally showed that immune sera and immunoglobulin with 219$ and antiviral agents for systemic use with 159$ consist the most important medication for PID patients during hospital admission (Table 2).

**Table 2:** Data analysis on cost of medications and drug in different types of primary immunodeficiency diseases

**Drug groups**	**Total Cost**	**D83.9**	**D81.9**	**D80.4**	**D80.2**	**D71**	**D82.4**	**D70**	**D84.9**
Anti-infective for systemic use	770.5±215.2	745.1±86.7	2005.2±396.1	502.2±63.3	78.6±7.1	497.3±108.7	176.9±53.1	160.8±27.8	181.7±42.1
*Beta-lactam antibacterials, penicillins*	59.9±17.9	22.1±6.4	140.2±28.1	1.3±0.1	0	47.3±18.1	53.1±17.6	0.3±0.08	58.0±13.9
*Other beta-lactam antibacterials*	91. 3±24.9	34.7±7.5	224.9±43.5	53.7±7.2	30.9±4.7	109.1±23.8	15.7±2.4	87.1±14.8	18.8±3.1
*Sulfonamides and trimethoprim*	40.9±18.5	10.9±2.9	120.0±35.5	86.0±19.2	7.4±0.6	17.1±2.7	20.0±5.9	0.09±0.02	5.3±1.4
*Macrolides, lincosamides and streptogramins*	24.4±9.9	7.2±1.4	55.0±18.2	73.7±16.2	3.9±0.08	27.3±4.0	0.6±0.1	17.9±4.6	6.0±1.0
*Aminoglycoside antibacterials*	3.4±1.8	0.6±0.2	10.9±3.7	0	0.1±0.04	2.4±0.06	1.0±0.5	0	0.6±0.2
*Other antibacterials*	102.7±49.0	29.4±8.7	311.2±96.7	11.2±2.5	22.5±3.2	43.8±8.9	39.7±13.1	54.5±8.8	32.8±8.9
*Quinolone antibacterials*	37.2±21.5	11.8±3.8	52.0±23.5	0	0.08±0.02	164.8±48.1	0	0	0
*Antivirals for systemic use*	159.6±73.3	0.9±0.3	76.5±21.4	0.4±0.1	6.2±1.0	78.8±18.8	3.5±0.7	0.7±0.2	4.5±1.2
*Antimycotics for systemic use*	30.7±12.9	0.5±0.02	0.7±0.2	0	0.1±0.04	0.1±0.03	0	0	0
*Antimycobacterials*	0.2±0.01	35.7±13.4	568.9±140.5	0	7.0±1.8	10.2±3.9	42.4±14.9	0	46.6±21.0
*Immune sera and immunoglobulin*	219.6±67.1	589.6±73.1	444.4±110.5	275.5±61.6	0	0	0	0	8.66±3.7
Drug for sensory organs	0.38±0.17	0	0.7±0.2	0.1±0.02	0.05±0.01	0.06±0.01	1.3±0.3	0	0.44±0.1
Dermatological agents	3.5±1.2	0.1±0.06	6.1±1.4	1.8±0.4	0.8±0.1	1.2±0.3	6.0±1.9	0.2±0.05	6.1±2.0
Drug for respiratory system	22.0±10.8	10.7±2.8	57.1±21.2	14.5±1.3	13.1±1.7	9.3±3.1	10.2±3.3	20.9±5.9	5.3±1.2
Drug for blood and blood forming organs	168.1±54.4	179.2±52.6	511.5±93.7	5.4±1.0	75.8±20.0	10.8±3.8	34.3±9.7	2.3±0.2	17.9±4.1
Drug for alimentary tract and metabolism	464.0±172.6	186.2±40.4	1407.4±330.1	132.2±26.8	53.2±7.4	178.6±31.7	88.1±19.3	230.4±60.2	166.6±34.2
Drug for nervous system	15.8±4.4	2.0±0.6	26.7±5.1	8.0±1.7	0.6±0.1	18.4±4.5	30.4±8.9	12.1±3.2	12.2±3.2
Drug for musculoskeletal system	0.6±0.05	0	0.17±0.01	0	0	0.18±0.07	0	0	0.06±0.05
Drug for cardiovascular system	18.1±8.8	0.8±0.3	25.9±8.7	0	0.2±0.05	2.6±0.9	106.4±23.3	5.3±1.4	1.1±0.2
Drug for antineoplastic and immunomodulating agents	16.3±7.0	0	24.5±7.7	0	0	0	9.8±2.2	100.2±19.5	11.8±5.1
Systemic hormonal preparations, excl. sex hormones and insulin	94.5±77.6	17.9±5.6	332.7±155.3	182.3±40.7	5.2±1.3	2.1±0.7	0.7±0.2	7.1±2.0	2.6±0.7
Various	10.8±3.0	6.5±2.2	24.7±5.1	0	0	12.0±2.2	0	17.6±3.5	4.6±0.9

D83.9: Common variable immunodeficiency, unspecified, D81.9: Combined immunodeficiency, unspecified, D80.4: Selective deficiency of IgM, D80.2: Selective deficiency of IgA, D71: Chronic granulomatous disease, D82.4: Hyper IgE syndrome, D70: Agranulocytosis, D84.9: Immunodeficiency, unspecified

### Drug costs in PID groups

Drug cost during admission consisted 4.6% to 28.1% of the total admission costs, based on type of PID; however, this variability was not statistically significant (F=0.52, *P*=0.81, Table 1).

Table 2 abstracted the cost of different groups of drugs, based on the PID disorders. Each drug group cost was evaluated internally for different PID to find which disease need more cost for special drugs. Cardiovascular system agents were used more in admission of chronic granulomatous disease patients in contrast with admission of common variable immunodeficiency (scheffe analysis: *P*=0.018) and hyper IgE syndrome (scheffe analysis: *P*=0.024). Combined immunodeficiency had higher rate of utilizing blood-forming drugs in comparison of chronic granulomatous disease (*P*=0.027). In the other hand, antineoplastic therapeutic agents were consumed in higher rate in common variable immunodeficiency cases rather than agranulocytosis and chronic granulomatous disease patients (*P*=0.011 and *P*=0.014, respectively).

### Effect of different variables on drug costs

**Fig. 1:**
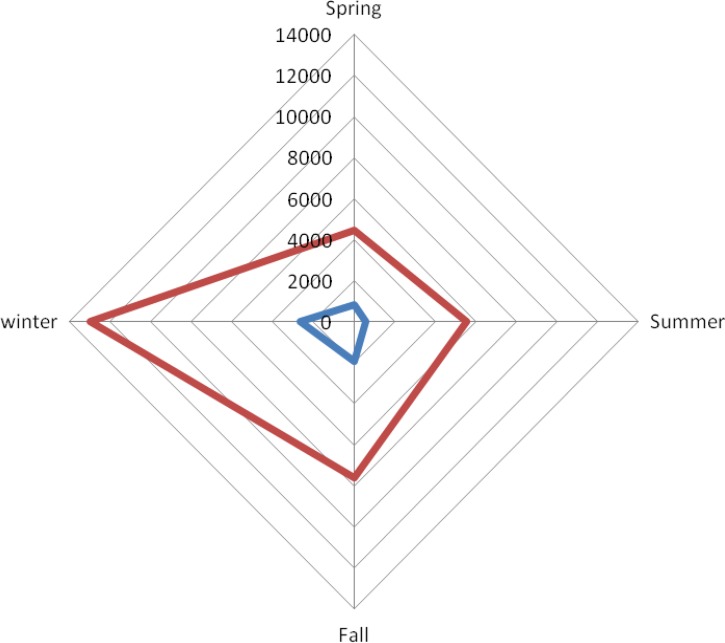
Cost of admission (red line) and drugs (blue line) of primary immunodeficiency patients during different seasons; spring (n=23), summer (n=24), fall (n=33), and winter (n=29)

Comparison of admission costs of PID in different seasons showed higher mean cost of admission (12940±4732.6$) and mean cost of drugs (2620.7 ±873.8$) in winter, but such differences were not significant (F=0.53, *P*=0.66; Fig. 1). There was no association between age of PID patients and the admission cost (r=−0.16, *P*=0.09).

## Discussion

There are limited data on admission and drug costs of PID treatment, though many studies accompanied about immunoglobulin costs (28–36). Although calculation of other drugs costs and modalities on PID is necessary, no study has been accomplished so far. Costs of medications only consist about one-fourth to one-third of total cost of PID patients during the hospitalization period.

Consideration of main cost drivers for admission of PID patients leads policy makers to provide a program to enhance patient outcomes and improve quality of life besides lowering costs. Making some structure and strategy, in the end, could be translated into critical cost saving to an organization.

Our findings showed the higher admission rate in the combined immunodeficient patients, while the predominantly antibody deficiency are the most frequent diagnosed group of patients based on the recently published report of national registry (22). Although this phenomenon is reasonable due to the nature of the immune defects in these patients comparing to other groups of patients, this group of disorder can be screened for newborns (facilitating finding of donor and prevention of side effect of immunization with lived vaccine) and can be cured by HSCT (37). Moreover current reports on the significant direct burden of PID can also indicate the necessity of fund allocation for molecular diagnosis for finding the genetic defects in the patients and subsequently on their relative for carrier detection and for helping by genetic consultation, prenatal diagnosis and/or preimplantation genetic diagnosis (38). This approach is not only according to the model for reduction of incidence and prevalence of autosomal receives disorders in the country with higher rate of consanguinity. However it can reduce the rate of X-linked disorders as in our study the majority of patients were male and this could be due to the presence of PID with X-chromosome inheritance (e.g. X-linked agammaglobulinemia, X-linked hyper IgM and X-linked severe combined immunodeficiency) in which carrier detection is essential in all female siblings and maternal aunts (1, 39).

Conformity of drug costs in the patients with the specific complication of each PID group and absence of correlation between age and admission cost suggests that the direct economic burden of PID disease forced in the long term in the affected children.

Two of every five PID patients are missing physician or hospital care, or failing to fill their prescription due to cost of disease (40). Therefore, finding the greatest consumable cost in PID cases and supporting such health cares with sort of health coverage and insurance may increase the therapeutic compliance of the patients with these chronic diseases and subsequently improve the number of routine follow-up visits and the quality of life.

The cost of anti-infective for systemic use was much higher than immune sera and immunoglobulin drugs in our series, which reflect the neglecting about the proportion of these agents in total drug costs of PID patients. However, pattern of this cost may differ between hospitalized patients and out-patients that received immunoglobulin products monthly (27).

Evaluated PID patients had annual mean cost of admission 7090.4$ and annual mean cost of pharmacotherapy of 1579.8$ per admission. The study by the Jeffrey Modell Foundation in the US estimated hospitalization cost of each PID patient about 16328$ annually. Moreover, hospitalization cost annually is about 22223$ in undiagnosed patient, which differs near to 7000$ with each other. Therefore, 40 billion $ saving in the US health care system per year by diagnosing people earlier and if that is cost of screening is about 5$ per each newborn baby (41). This could show the importance of early diagnosis in preventing complications and hospitalization in PID patients. Beside this estimation, the cost in our country seems to be much lower than the US, but it may be underestimated because of the economic conversion of cost units and may be due to environment of this study, which held only in a children’s hospital with lower needs to drugs dosage.

It appears pharmacotherapy in these patients is a major contribution to the direct costs of PID; this does not diminish the other expenses like hospital facility costs, and patient evaluating lab and diagnostic test. Although as of high risk of infection, antibiotics have the most significant contribution in drug costs of PID patients; this study does not show any significant variability between antibiotics and other drug groups. According to the Jeffrey Modell Foundation reports, antibiotic therapy for each patient in each episode per day is about 4.25$ with 309$ annual cost per patient (41).

Despite these findings, previous studies only focused on treatment by immunoglobulin agents’ costs, especially in PID patients that need this drug as permanent members of the treatment. An annual cost of 15.470$ were estimated for intravenous immunoglobulin (IVIG) replacement therapy in PID patients, which includes both costs of admission and outpatient administration. Moreover, regular treatment with IVIG can reduce cost of nursing and hospitalization and may save 2000–5000$ per patient per year (42).

More than half of PID patients faced some problems such as avoiding hospitalization, receiving insufficient dose of IVIG, or not being visited by specialist, because of the medical care costs. In addition, at least one fourth of PID individuals have complaints about insurance problems (43).

Although this study focus on the measurement of baseline costs of PID, however, this preliminary data is required for further health policymaking studies inducing measurement of efficiency, effectiveness, productivity or performance on new diagnostic or therapeutic methods.

## Conclusion

This study highlights the need for more well-designed prospective and retrospective studies on disease-specific economic burden and costs analysis. It could provide a good chance for health policy makers to plan for PID patients and allocate some fixed budget for early diagnosis of patients and prevention of further complications that exceed the medical care costs. Making some structure and strategy, in the end, could be translated into critical cost saving to an organization.

## Ethical considerations

Ethical issues (Including plagiarism, informed consent, misconduct, data fabrication and/or falsification, double publication and/or submission, redundancy, etc.) have been completely observed by the authors.
